# On Excessive Sweating

**Published:** 1869-04

**Authors:** Arthur Wynne Foot


### ON EXCESSIVE SWEATING.

By ARTHUR WYNNE FOOT, M.D., F.K., & Q.C.P.

Among the more remarkable instances of general and excessive, that is, visible sweating, are the colliquative perspirations attending the venereal, cancerous, or phthisical cases, the drenching sweats of over-lactation, of the terminal stage of the normal ague paroxysm, the sweatings or crisis of rheumatic fever, and those occurring about the time the ovaries become effete. The exhausting effects of those obviously unnatural cutaneous discharges have, as is just and right, caused more anxious search to be made for any means of moderating or controlling them, than of explaining their etiology. It appears as if the causes of general profuse sweating might be referable to two causes (perhaps to but really one): to the circulation of a poisonous blood, and to depression of the vital powers; that is, to diminished energy of the sympathetic system. The condition of the temperature, pulse, and skin at the termination of an ague paroxysm points to a paralyzed and dilated condition of the arteries; the hepatic, splenic, or thyroidal enlargements may result from excessive flow of blood to these parts from the same cause. The earlier stage of poisoning by malaria appears very different from the latter one, the cold, wrinkled, shivering surface contrasting with the hot, flushed, sweating one; but in other poisons of vegetable origin, as in opium, the earlier effects are stimulant and very different from the later ones, and the phenomena observed in the first stage correspond to those which attend experimental stimulation of the sympathetic nerve. Cruveilhier remarks, that of the three stages of a paroxysm of intermittent fever, that of sweating is the most constant. Some attacks may be without the cold stage, others without the hot stage; but it is very rarely that the sweating stage is absent; he has recognized an irregular ague by the nightly occurrence of profuse sweating amenable to quinine. There is reason to believe that the perspirations of rheumatic fever, although they may seriously compromise the comfort of the patient, and he may be sensible of but little relief from pain by them, yet serve to remove from the body much of the *materies morbi* which is the exciting cause of the symptoms. In phthisis, profuse perspiration, although generally a symptom of very advanced disease, may be present at a period when both auscultation and percussion give only dubious information as to the condition of the lung, and in such case it will be found generally to be disseminated tubercle from which the individual is suffering. Cruveilhier records a remarkable case of this kind, which, as far as physical diagnosis was concerned, remained undetermined up to the person's death, but which was marked from the very first by excessive night-sweats, accompanied with rapid emaciation and profound debility. Regarding the colliquative sweating of phthisis as a consequence of vaso-motor paralysis resulting from constitutional exhaustion, it is not difficult to appreciate the value, although often but temporary, of nutritious diet, and such medicines as cod-liver oil. In support of the view that phthisical sweatings are the natural result of the depressed condition of the vascular system of the patient, are the observations that these perspirations usually come on when the person falls asleep, and more frequently during night sleep than day sleep; it has also been shown by Dr. Edward Smith, that in cases of phthisis, the subsidence of the rate of pulsation during the night is much greater than in health, and that the greater difference between the day and night rate in phthisis than in health is less due to the increased elevation of the pulse during the day than to the great subsidence of it through the night. The usual time for the occurrence of perspiration in typhoid fever is in the night, the skin in the daytime being usually dry; and according to Traube's researches, the critical evacuations in typhus fever, and among them perspiration, are always preceded by a considerable fall in the pulse. In many other cases depression of the heart's action is a well-known cause of perspiration—for example, those attending the syncopal state which the inexperienced smoker falls into, the sweating which attends sea-sickness, extreme purgation, the exhibition of tartarized antimony, and the effects of terror. The remedies which prove serviceable in the majority of cases of excessive general sweating are such as tend to remove depression of the heart's action, and act as tonics on the vaso-motor nerves. The difference in gravity of import between local and general sweatings appears to be very great, because, although a partial sweating may excite more alarm and closer attention from the greater rarity of its occurrence, yet it does not appear that it is in most cases indicative of by any means as serious nervous disturbance as are the general sweatings.

For the relief of sweatings of the hands, Dr. Druitt has suggested the thorough application of the hottest water that can be borne without pain to the offending parts until they are red hot, and tingling as if scalded. This treatment, the author states, sometimes appears to aggravate the affection. Hebra recommends the frequent local use of a solution containing one drachm of tannic acid mixed in six ounces of alcohol; this liquid should be rubbed into the part several times a day, and the skin must not be wiped afterwards: a little powdered asbestos is to be sprinkled on it while still wet, and with this the part is to be rubbed till it is dry. From statistics, he finds that this complaint affects the young as well as the old, both males and females, rich and poor, those who are of cleanly habits and those who are dirty, persons who are in good health and those who suffer from other maladies.—*Dublin Quarterly Journal of Medical Science, May.*—*Ranking's Abstract.*

